# Empirical evidence of the effect of school gathering on the dynamics of dengue epidemics

**DOI:** 10.3402/gha.v9.28026

**Published:** 2016-01-06

**Authors:** Carlos M. Hernández-Suárez, Oliver Mendoza-Cano

**Affiliations:** 1 Facultad de Ciencias, Universidad de Colima, Colima, México; 2 Center for Health and the Global Environment, Department of Environmental Health, Harvard TH Chan School of Public Health, Boston, MA, USA; 3 Facultad de Ingeniería Civil, Universidad de Colima, Coquimatlán, Colima, México

**Keywords:** dengue, mosquito, school, community intervention, *A. aegypti*, Colima

## Abstract

**Introduction:**

Dengue fever is an important vector-transmitted disease that affects more than 100 countries worldwide. Locations where individuals tend to gather may play an important role in disease transmission in the presence of the vector. By controlling mosquitoes’ breeding places, this study aims to analyze the effect of reducing transmission in elementary schools (grades 1–9) on the dynamics of the epidemic at a regional level.

**Materials and methods:**

In 2007, we implemented a massive campaign in a region of México (Colima state, 5,191 km^2^, population 568,000) focused on training janitors to locate and avoid mosquitoes’ breeding places, the objective being to maintain elementary schools free of mosquitoes.

**Results:**

We observed 45% reduction in dengue incidence compared to the previous year. In contrast, the rest of Mexico observed an 81% increase in incidence on average.

**Discussion:**

Costs associated with campaigns focusing on cleaning schools are very low and results seem to be promising. Nevertheless, more controlled studies are needed.

## Introduction

Dengue fever, a disease transmitted by the mosquito *Aedes aegypti*, is endemic to more than 100 countries with an estimated 50 million dengue infections worldwide every year (1). Although there is a lot of evidence on the importance of schools and other gathering places on the transmission of airborne diseases like influenza (2–4), it was only recently that Chao et al. (5) found that school opening dates predict influenza outbreaks in the United States. Whereas gathering is unarguably a driving factor on the transmission of airborne diseases, its effect is less obvious for vector-transmitted diseases. In a study in Thailand, Endy et al. (6) concluded that most dengue virus transmission occurs within the community or the schools, and there is evidence that transmission in schools plays an important role in the progression of dengue epidemics (7), showing a strong association between a rise in the dengue seroconversion rate and the start of formal schooling. However, neither its contribution to the epidemic size nor the effect of campaigns focused on reducing transmission at these places have ever been quantified.

In México, elementary schools are left unattended for almost 2 months during the summer break; many of them become a suitable environment for mosquitoes. After the break, students in their first 9 years of education return to classes. These students account for approximately 25% of the population in México, without considering high school or higher educational levels. Thus, a great deal of the population suddenly increases their exposure to the vector.

**Fig. 1:**
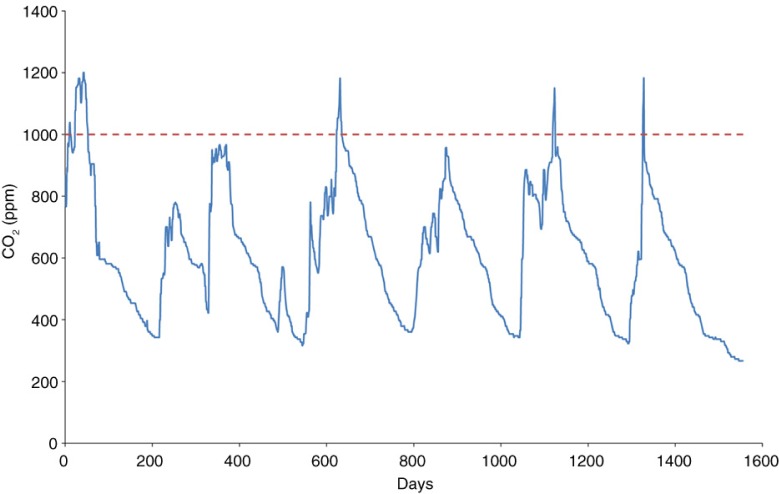
Carbon dioxide measurements in a classroom during a given week. The dotted line at 1,000 ppm level shows the recommended ASHRAE compliance for CO_2_ levels for acceptable indoor air quality (see Ref. 8).

*A. aegypti* locates humans and other animals mainly through CO_2_, which reaches high concentrations in small environments. A study by Cruz (8) showed that CO_2_ concentration levels in classrooms exceeded the recommended level of the American Society of Heating, Refrigeration and Air Conditioning Engineers (ASHRAE) for acceptable indoor air quality (Fig. 1). It is therefore easy to classify schools as potentially risky environments, at least under the strong presence of *A. aegypti*.

Within endemic zones in México, mosquito-free schools are difficult to find. Janitors are generally trained to maintain classrooms clean, but they do not see the presence of mosquitoes as a health threat. It is not part of their job description or training to be able to locate, clean, or even report breeding sites. Many objects and areas in schools serve as artificial and natural containers where water may accumulate (e.g. bottles, tires, roofs, and plastics from recycling programs). We estimated that if one of every four individuals of a population attends school 5 h daily 5 days a week, where the contact rate increases by a factor of 30, then the population average contact rate duplicates (see Appendix 1).

There have been other efforts to control epidemics via educational tools, among these, Espinoza et al. (9) analyzed the role of educational campaigns on larval indices in a study in the state of Colima (México). In that prospective study, significant differences were found in several larval indexes between households that participated in the educational campaign and those that did not. In Sri Lanka, Jayawardene et al. (10) implemented a community intervention campaign involving students in grades 7–9 that reduced several measures of larval incidence following the intervention. This campaign was implemented in urban and rural areas but did not focus specifically on schools.

Evidence of an increase in the infection rate immediately following the beginning of the school year is provided by available data. Generalizing the theory developed by Watts and Strogatz (11), dengue transmission should increase when new links between individuals are established. In our case, this occurs at the beginning of the school year in schools containing the vector. This line of reasoning is what motivated the campaign that we will be described later.

**Fig. 2:**
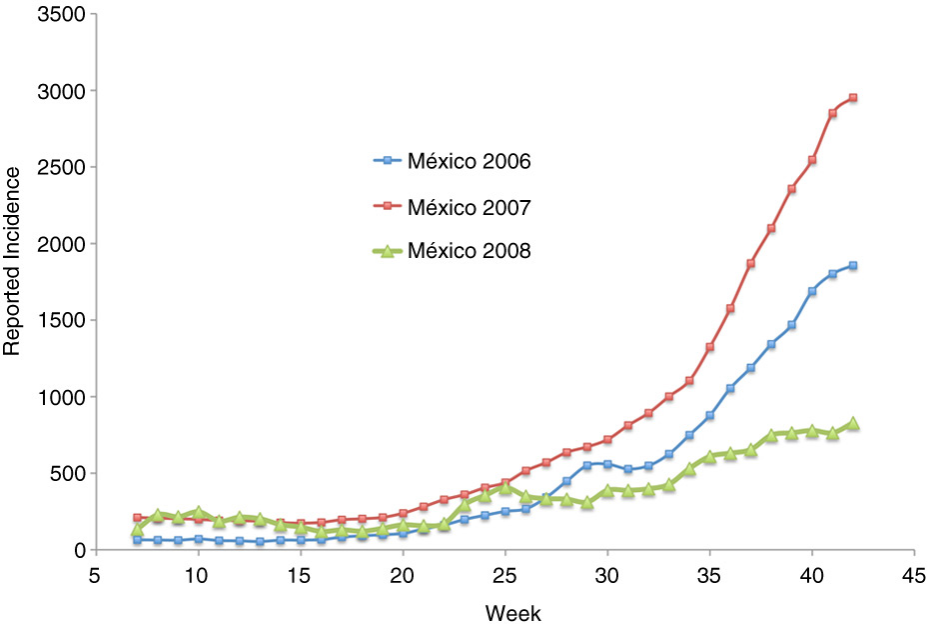
Reported dengue incidence in México 2006–2008. Source: CENAVECE (12). Classes begin around week 33.

Figure 2 shows the 3-week running average incidence of a dengue fever epidemic; this includes both classic and hemorrhagic cases in Mexico for the years 2006–2008. This official data were obtained from the National Center for Epidemic Surveillance, CENAVECE (12). Only confirmed cases were reported. The original data set consists of the date in which the patient disclosed presenting symptoms (fever, headache, and eye pain). We subtracted 3 days in an attempt to estimate the beginning of the infection (i.e. the mosquito bite). We can see two points at which the rate of growth of the epidemic seems to increase: the first is at the beginning of the rainy season (around week 22), and the other around weeks 31–33. While the start of the rainy season varies widely in México, the beginning of classes is the same for all elementary schools in México every year (the beginning of the third week of August).

**Fig. 3:**
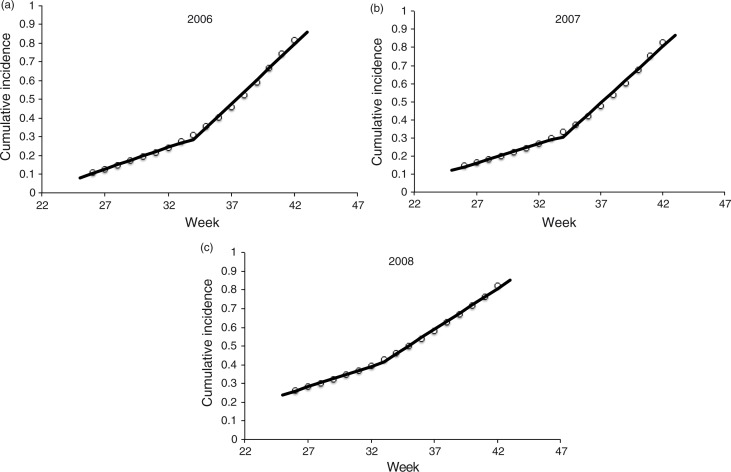
Normalized cumulative incidence of dengue cases for México in the years (a) 2006, (b) 2007 and (c) 2008. Shown are the two lines that yielded the highest *R* ^2^ using data from weeks 26 to 42. Source: CENAVECE (12).

We model dengue transmission with a *Susceptible–Infected* mathematical model of infection (13), where the infection rate when there are *I* infected and *S* susceptible is λ *I S*/*N*, where λ is the per capita infection rate and *N* is the population size. If we assume the number of susceptible individuals is large, then *S*/*N* tends to 1 and the infection rate can be approximated with the usual linearization λ*I*. This implies that the accumulated number of infected individuals at the next unit of time *F*(*t+1*) is related to the current number of accumulated infected as *F*(*t+1*)≈(λ+1) *F*(*t*) (see Appendix 2). Thus, a plot of *F*(*t+1*) versus *F*(*t*) should be linear, unless there are changes in the per capita contact rate. Figure 3a–c shows the normalized cumulative incidence using México's data from weeks 26 to 42 for years 2006–2008.

The two straight lines fitted to each of Fig. 3a–c were adjusted to every subplot in an attempt to detect a change in per capita contact rate. To adjust the two lines, we vary the break-point until the weighted *R* ^2^ was maximized using the number of data values in each segment as weights. In each case, (λ+1) is the slope of each of the adjusted lines. During the years 2006 and 2007, the change in slope occurred between weeks 33 and 34. In the year 2008, the change occurred between weeks 32 and 33. The cumulative incidence at the break-point each year was 27.4, 29.8, and 39.5%, respectively. The respective increase in the infection rates immediately following the break-point was 2.7, 2.9, and 1.9, respectively. Additionally, the break-point each year occurred at weeks 34, 34, and 33, respectively.

It is important to note that the data on infection occurrence are inaccurate. Furthermore, the location of the break-point strongly depends on the weeks selected (the break-point tends to increase if we increase the lower limit for the data set used to fit the lines). Nevertheless, it is of interest to explore the existence of a boosting factor at around week 33. To this end, we decided to implement a campaign focusing on clearing all elementary schools in the state of Colima of mosquito breeding sites.

## Experimental section

**Fig. 4:**
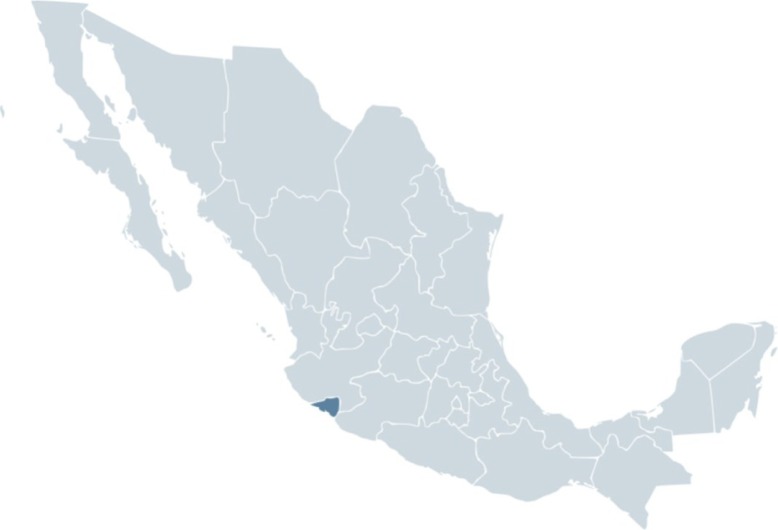
Map of México showing the location of the state of Colima.

The state of Colima (population 568 K, 5,791 km^2^, see location in Fig. 4) is one of the states with a higher dengue incidence in México. The average temperature is 28°C ranging from 12°C to 38°C, with an average rainfall of 983 mm. In 2007, we implemented a special statewide campaign focusing on teaching janitors of elementary schools to locate and get rid of mosquito breeding places in their schools prior to the beginning of classes, in 482 schools with 75,000 students (average school size 120±131). We decided to work with janitors in an attempt to be the least invasive possible. Although a campaign including teachers and students would very likely be more efficient, our goal was to test the effectiveness of simple actions on the dynamics of dengue in a given region. This way, other communities would be able to easily apply the same campaign, if effective.

The state of Colima is divided into 10 counties, and the Department of Education was responsible for organizing the attendance of each county's janitors to a talk; this talk lasted 2.5 h on average. The attendees of each county were divided into groups of 30 individuals on average. All groups were trained simultaneously. The talks were carried out in the school of the county better suited to it. For instance, it was necessary to have enough classrooms to be able to accommodate up to 100 janitors and windows that could be covered to darken the rooms for better image display. First, a short survey with 10 questions was applied to every group before the talk. The same survey was also applied after the talk, the purpose being to compare the participants’ knowledge on dengue transmission and control before and after the presentation. Using slides and a projector, the training included about 1 h of sharing information including the life cycle of a mosquito, the transmission of dengue and symptoms. The rest of the time (approximately 1.5 h) janitors were shown typical places inside schools all over the state where the mosquito could breed and live. In every meeting, we had the assistance of personnel from both the department of health and education. Participants posed a lot of questions regarding what to do in specific situations, for instance, many janitors complained that their tasks included keeping classrooms clean as well as other administrative offices, but that they had not been previously trained to locate and clean mosquitoes’ breeding places, nor had they been given the utensils to do so. In general, there was a favorable disposition to participate. Special emphasis was placed on empowering them as important members of a public health campaign.

There were a total of 1,284 attendees at the meetings that included personnel from 91% of schools in the state of Colima, including both public and private schools. The remaining 9% were mostly private schools. The unrepresented schools were due mainly to misinformation or to the fact that some schools simply lacked a janitor. In many schools, one or more representatives from the parents association were present. However, no professors were present since the campaign was carried out during the summer break. During the talk, janitors discussed and exchanged information among themselves on what could be done to reduce the number of breeding places. There were two common complaints throughout the state: the first related to the impossibility of getting rid of old furniture and other equipment (mainly plastic chairs) since regulations require paperwork to dispose of these materials. Therefore, such objects would typically remain on the school premises for more than 2 years. The second complaint was regarding the lack of communication between janitors and certain authorities that in the past apparently disregarded some janitors’ request for tools to repair water leaks or to cover places where water might accumulate during the rainy season. We took these complaints to the highest level in both the education and health departments. Additionally, we visited the Major's office in every county to discuss complaints pertaining to trash collection. Since many janitors were not present during the summer break, there was a second round of visits to every county; 24.7% of the total attendees were present during the second round. Our main impression during the campaign was that a huge reduction to breeding sites in schools could be achieved with relatively little effort from janitors and authorities.

## Results and discussion

According to the survey, 82.3% of janitors believed the school they worked in posed no risk of dengue transmission prior to the meetings compared to 22.7% after the meetings. Traditionally, extensive campaigns by health authorities have focused their attentions on cleaning up homes with no mention made to public places. Because of this, we believe the high level of confidence exhibited by custodians prior to the meetings is due mainly to disinformation. In addition, we believe the low level of formal education among janitors (an average of 7.5 years of school education) played an important role.

**Fig. 5:**
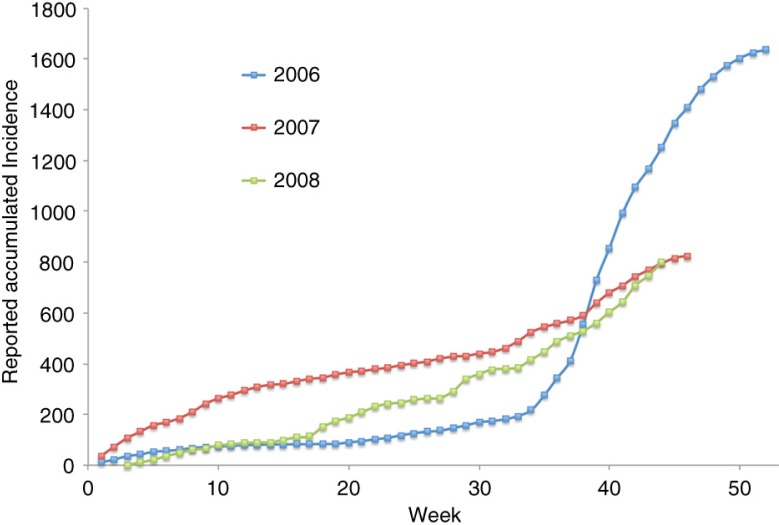
Reported accumulated dengue incidence in the state of Colima for the years 2006–2008. Source: National Center for Epidemic Surveillance. The beginning of the school year is at week 33. Cleaning campaign in 2006 started at week 22. Source: CENAVECE (12).

**Fig. 6:**
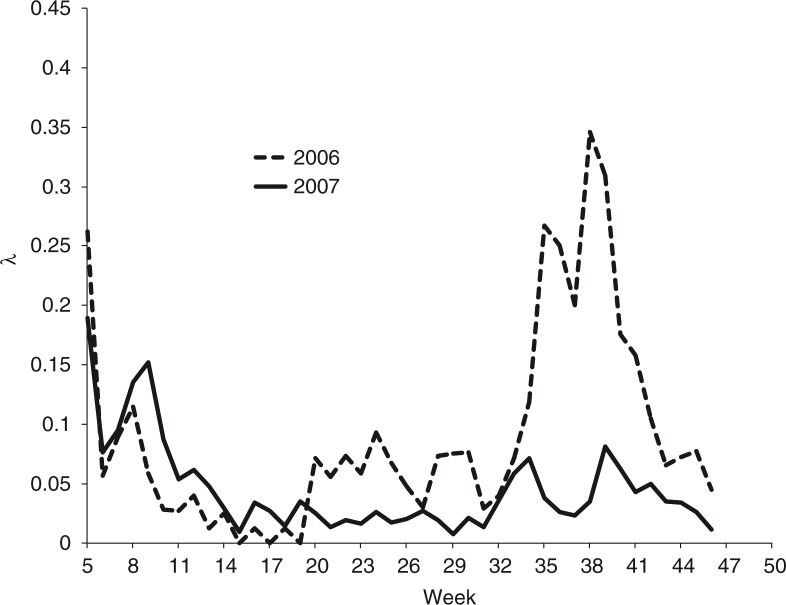
The per capita contact rate for the dengue epidemics in the state of Colima during years 2006 and 2007. The per capita contact rate at week *t* is computed as *F*(*t*+1)/*F*(*t*)−1, where *F*(*t*) is the accumulated incidence at time *t*. Source: CENAVECE (12).

By the end of 2007, we observed a reduction in the size of the jump in per capita infection rates right at the beginning of the school year compared to the previous year (see Fig. 5). Figure 6 shows a plot of λ, the per capita infection rate for both years (13). The jump in the per capita infection rates is more noticeable around week 33. By week 35, 2 weeks after the beginning of classes, the per capita infection rate in 2007 was 0.03, compared to 0.26 in 2006.

Table 1 shows a comparison of incidence rates in 2006 and 2007 for two moments in time: at week 28 (after the beginning of the rain season) and at the end of the year, week 52. We can see that during week 28 of the year 2007, the increase in incidence for the state of Colima compared to 2006 (2.93) was about the same as for the rest of Mexico (3.54). Nevertheless, by week 52 the increase in incidence in the rest of Mexico was 1.81, whereas for the state of Colima it decreased to 0.55.

**Table 1:** Ratio of the cumulative incidence in 2007 to that in 2006 at weeks 28 and 52

	Week 28	Week 52
Colima state	2.93	0.55
Rest of México	3.54	1.81

Comparison of Colima versus the rest of Mexico. Source: CENAVECE (12).

Unarguably, the dynamics of dengue in the state of Colima in 2007 was not only driven by the school-oriented campaign. There were other factors such as the already existing campaign by health authorities at the population level, which consisted in the use of communication media (mostly radio and newspapers) to persuade people to locate and clean mosquito breeding places in their homes. In addition, other preexisting campaigns were the door-to-door visits by health personnel and the occasional spraying in sectors suggested by vector surveillance. Nevertheless, these campaigns were also implemented in 2006, leaving the work with custodians as the main difference in the year 2007. Prior to the campaign, we could account for the large number of water depositories in schools that would become breeding places for mosquitoes. In some schools, the amount of mosquitoes was unbearable. The authors had the opportunity to show the pictures of these schools and their conditions in a general meeting of health secretaries from all Mexican states where dengue is endemic. The meeting was organized by the National Center for Epidemic Surveillance (CENAVECE) in Villahermosa, Tabasco, in 2008. The general consensus was that the school's situation in other states was no different.

**Fig. 7:**
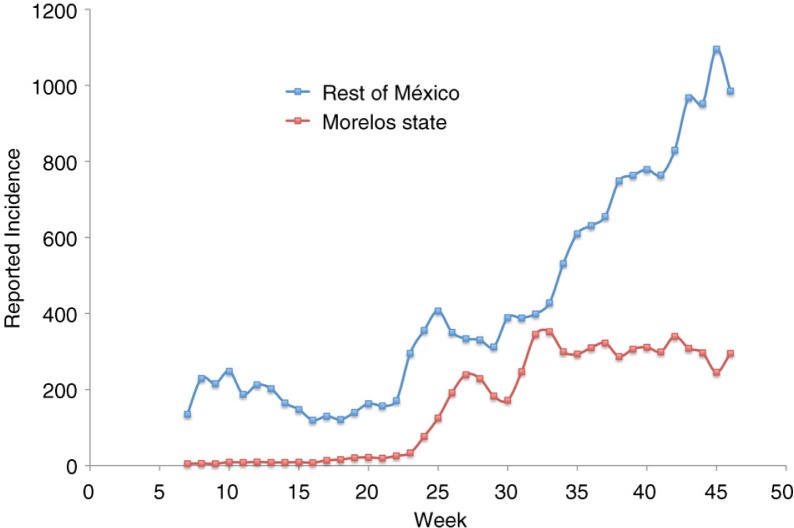
Three-week running average of dengue incidence in Morelos state and the rest of México in 2008. School began at week 33 in México except in Morelos where it was delayed by 3 months. Source: CENAVECE (12).

The possibility of carrying experiments focused on reducing the number of individuals gathered in schools is limited, because it is very difficult to suspend gathering in schools for long periods of time. Nevertheless, in 2008 we had the opportunity to study the evolution of the epidemics when crowdedness in schools was dramatically reduced due to a teachers’ strike in the state of Morelos (population 1.6 M). During that year, classes began in the third week of August. The 3-month delay extended the gathering of more than 400,000 students from elementary schools until December 2008. Figure 7 shows the 3-week running average of the incidence for the state of Morelos compared against the rest of México. This plot shows that in 2008 Morelos state kept a constant individual contact rate during the epidemics, and thus no jump in the incidence rate at the beginning of classes was observed. Meanwhile, the rest of México showed a noticeable jump around week 33. Studies to reconstruct the chain of infection may help delimitate the role of dengue transmission in schools. Aside from elementary schools, many families leave their children who are too young to attend elementary school at hundreds at nursery or pre-school houses, most of them are ordinary large houses that have been conditioned to serve that purpose. While our campaign did not include these locations, it is our belief that these may also be important gathering places where dengue transmission takes place.

## Conclusions

The potential effects of reducing transmission at the school level and other gathering places are worthy of consideration, especially taking into account that the education of personnel associated with the maintenance of schools is inexpensive (administrative structures exist to do so). The relation cost/benefit seems to be promising.

We conclude that the described intervention gave an empirical evidence to avoid dengue transmission in schools. The relationship of school activities and the high incidence of dengue can be useful for health decision-making especially in the development of resource allocation programs for vector control in other countries.

## Appendix 1

In the Small world phenomena (11), the degree of contact of a population is higher than it looks and closely resembles that of a random mixing where all susceptible individuals have equal risk of becoming infected. Let *R*0 be the basic reproductive number when the population does not gather. If a fraction *f* of individuals attends school 5 h a day five times a week and if the contact rate increases by a factor of *k*, *R*0 changes to *R*0* and the overall contribution of the people who gather to this *R*0* is

kR0f(5/7)(5/24),

whereas the contribution of those who do not gather is

R0(1-f(5/7)(5/24)).

Adding both terms, dividing by *R*0 and letting *f*=1/4 and *k*=30 yields 2.07, which is the number of times *R*0* is larger than *R*0.

## Appendix 2

We use a susceptible–infectious (SI) model (13) where the growth rate of the number of infections at time *t* is *I*′(*t*)= λ*I*(*t*). Since the model is an SI we have *F*(*t*)=*I*(*t*). Using a discrete approximation, the relationship between the cumulative incidence at time *t* and that of time *t*+1 is

[F(t+1)-F(t)]/F(t)=λ,

where λ is the per capita infection rate per time unit. It immediately follows that

F(t+1)/F(t)=λ+1.
