# Corrigendum: Empirical evidence of the effect of school gathering on the dynamics of dengue epidemics

**DOI:** 10.3402/gha.v9.31213

**Published:** 2016-02-18

Regarding the paper titled: ‘Empirical evidence of the effect of school gathering on the dynamics of dengue epidemics’ by Carlos M Hernández-Suárez and Oliver Mendoza-Cano

Published in Global Health Action (section “Original Articles”) 06 Jan 2016. **Citation**: Glob Health Action 2016, 9: 28026 - http://dx.doi.org/10.3402/gha.v9.28026

In the Results and Discussion section, in Figure 5, the legend reads “Cleaning campaign in 2006 started at week 22.” it should say “Cleaning campaign in 2007 started at week 22”.

The legend currently reads:

Fig. 5. Reported accumulated dengue incidence in the state of Colima for the years 2006–2008. Source: National Center for Epidemic Surveillance. The beginning of the school year is at week 33. Cleaning campaign in 2006 started at week 22. Source: CENAVECE (12).

The legend should read:

Fig. 5. Reported accumulated dengue incidence in the state of Colima for the years 2006–2008. Source: National Center for Epidemic Surveillance. The beginning of the school year is at week 33. Cleaning campaign in 2007 started at week 22. Source: CENAVECE (12).

